# A three-domain perspective on teacher–student relationship and bullying victimization in Chinese schools: a serial mediation

**DOI:** 10.3389/fpsyg.2026.1820612

**Published:** 2026-07-22

**Authors:** Yifan Miao, Yi He, Renjie Zhang, Xuchu Chen

**Affiliations:** 1 Institute of International Studies, Wenzhou Business College, Wenzhou, China; 2 Institute of Higher Education, Wenzhou University, Wenzhou, China; 3 Wenzhou Foreign Language School, Wenzhou, China

**Keywords:** bullying victimization, school belonging, school exclusion, teacher emotional support, teacher instructional support

## Abstract

**Introduction:**

Guided by the Teaching Through Interactions framework, this study examined how teacher emotional support, teacher instructional support, and disciplinary climate were related to students’ school bullying victimization, focusing on the serial mediating roles of school belonging and exclusion.

**Methods:**

A total of 798 students aged 12–16 years in eastern China were surveyed using the PISA-adapted scales to measure the domains of teacher–student relationship, school belonging, school exclusion, and bullying victimization. Structural equation modeling was used to assess direct and indirect pathways.

**Results:**

Teacher emotional and instructional support were positively associated with school belonging, which in turn was related to lower exclusion and reduced bullying victimization. Disciplinary climate was indirectly linked to less bullying, primarily through lower exclusion.

**Discussion:**

These findings clarify how multidimensional teacher–student relationship shapes bullying through school belonging and exclusion in Chinese schools. These findings extend social-ecological and interactional perspectives to the Chinese context and highlight practical levers for prevention, including strengthening teacher emotional and instructional support, fostering a fair disciplinary climate, and addressing school belonging and exclusion as proximal mediators and intervention targets.

## 1 Introduction

School bullying is a widespread social problem within educational contexts (Harris and Petrie, 2003), with significant implications for the well-being of children and adolescents (Hong and Espelage, 2012). According to a UNESCO report in 2019 covering 144 countries and territories, almost one in three students (32%) had been bullied at school at least once in the previous month (UNESCO, 2019). School bullying refers to intentional and repeated aggression toward a student perceived to have less power, perpetrated over time by one or more peers (Olweus, 1993). Previous studies have demonstrated that school bullying can lead to serious consequences for all parties involved, particularly for bully-victims. Students who are frequently bullied may suffer physical, verbal, or relational harm (Kennedy, 2020) and are more likely to report heightened stress, low self-esteem, loneliness, depression, anxiety (Gini and Pozzoli, 2009; Chu et al., 2019; Estévez et al., 2019) and symptoms of posttraumatic stress (Xu et al., 2023). In addition, bullying victimization has been linked to externalization problems such as poor academic performance (Riffle et al., 2021), social withdrawal (Choi et al., 2022), engagement in addictive behaviors (Richard et al., 2020), and even increased risks of self-harm and suicide (Van Geel et al., 2014; Peng et al., 2020; Ge, 2025). Although bullying is a global phenomenon, its expressions and underlying mechanisms often vary by sociocultural context. Given these profound impacts, identifying modifiable school-based relational and environmental factors that reduce bullying risk is a critical research priority.

In China, the educational setting is strongly shaped by collectivist values, where individuals are connected to close-knit social groups and place great importance on group harmony and collective interests (Huang et al., 2013; Smith and Robinson, 2019). This cultural context may heighten sensitivity to group norms and exacerbate peer exclusion for students who deviate from collective expectations (Huang et al., 2013). Moreover, the strong emphasis on academic achievement can unintentionally overshadow students’ socioemotional needs, leading teachers and parents to pay insufficient attention to bullying experiences (Huang et al., 2013; Xing et al., 2023). Recent large-scale evidence from China further shows that bullying victimization is associated with a wide range of psychological difficulties, with more severe victimization linked to substantially higher psychological risk (Zhao et al., 2024).

This study focuses on secondary school students in Wenzhou, a southeastern Chinese city where boarding is common and parental absence due to work-related migration is frequent. In this context, teachers often play a central role in students’ academic and socioemotional lives (Mariati and Silahuddin, 2024). Building on this context, we conceptualize the teacher–student relationship in terms of three domains: teacher emotional support, teacher instructional support, and disciplinary climate, and explore whether these domains are linked to school bullying victimization through serial mediation involving students’ school belonging and school exclusion.

### 1.1 Teacher–student relationship and school bullying victimization

With reference to the social-ecological framework (Bronfenbrenner, 1979), school bullying victimization is conceptualized as a social phenomenon resulting from the dynamic interplay between individual and contextual factors (Hong and Espelage, 2012). It always arises within the social environment in which it occurs (Williford et al., 2019). Within this framework, schools represent a critical microsystem, second only to the family context (Bronfenbrenner and Morris, 1998). In educational settings, teachers play a pivotal role, significantly influencing both the instructional and emotional climates of the classroom through daily interactions with students (Hamre et al., 2013). Thus, the school environment provides an important context in which teacher–student relationships may shape students’ feelings of safety, belonging, and vulnerability to peer victimization.

Attachment theory suggests that children seek proximity and contact with responsive figures, especially when their emotional security is threatened (Bowlby, 1982). Although Bowlby’s original theory was developed in relation to early child–caregiver bonds, later educational research has extended this perspective to school settings to explain how significant adults may provide security-related support for students. While parents are typically considered primary attachment figures, increasing attention has been given to the role of teacher–student relationship in school settings through the lens of attachment theory, particularly following Pianta’s (1992) seminal work. In the present study, teachers are not conceptualized as replacements for parents, but as school-based adults who may perform attachment-related functions, particularly for students experiencing stress, exclusion, or peer-related threat. In this context, teachers can be regarded as temporary or *ad hoc* attachment figures that provide care and guidance to multiple students simultaneously (Zajac and Kobak, 2006; Verschueren and Koomen, 2012). As safe havens in such relationship, teachers help students regulate feelings of insecurity and stress, ultimately restoring their sense of emotional security (Spilt and Koomen, 2022). This perspective remains relevant for middle school students, whose social world extends beyond the family and whose sense of security is increasingly shaped by relationships with teachers and peers in school. Previous studies have demonstrated that high-quality teacher–student relationship is linked to reduced school bullying victimization (Sulkowski and Simmons, 2018; Thornberg et al., 2022; Ten Bokkel et al., 2022; Lin et al., 2025). However, mixed findings also exist (Hamel et al., 2021; Demol et al., 2022), highlighting the need for further exploration into the specific components of teacher–student relationship that may be particularly protective against bullying.

In line with this perspective, recent research has increasingly focused on the role of teacher support as a specific aspect of teacher–student relationship, emphasizing its protective role against school bullying victimization (Kim et al., 2021; Thornberg et al., 2024; Guo et al., 2024). Many studies have concentrated on the emotional dimension of teacher support, which is defined as students’ perception of teachers and other school staff members as supportive, respectful, and willing to assist (Cornell et al., 2015), or as teachers responding to students’ social and emotional needs by demonstrating respect and care (Kim et al., 2021). Emotional support from teachers has been linked to increased peer acceptance (Hendrickx et al., 2017) and a reduced likelihood of bullying victimization (Guo and Zhao, 2019).

In contrast, the role of instructional support in relation to school bullying remains underexplored. Teacher instructional support refers to the active engagement of teachers in fostering students’ academic growth through consistent encouragement, instructional guidance, and assistance with tasks such as classroom learning, homework, and academic decision-making (Patrick et al., 2007; Granziera et al., 2022). Although this form of support has rarely been studied in the context of school bullying, it has been associated with other behavioral outcomes, particularly adolescents’ externalizing problem behaviors (Zhu et al., 2025). Furthermore, research has established that academically successful students are less likely to engage in problem behaviors (Wang and Fredricks, 2014; Burns and Becker, 2022). These findings suggest a potential link between teacher instructional support and reduced school bullying, although the underlying mechanisms remain unclear.

Guided by the Teaching Through Interactions (TTI) framework, which identifies emotional support, instructional support, and classroom organization as three core domains of teacher–student interactions (Hamre et al., 2013), we conceptualize the teacher–student relationship in this study as a three-dimensional construct. Consistent with our focus on student perceptions, classroom organization is operationalized as disciplinary climate, defined as students’ perceptions of rule consistency and stability and of how teachers respond to behavioral issues (Cheema and Kitsantas, 2014). From a social control perspective, it also involves how students internalize the school’s norms and values, aligning their behavior with these expectations (DiPrete et al., 1981). Empirical studies emphasize the significance of disciplinary climate for student motivation and academic achievement (Ma and Williams, 2004; Edman and Brazil, 2007).

A positive effect of climate level is usually expected when its strength is high (Schneider et al., 2017). When students perceive their learning environment as fair and well managed, they report higher engagement and more positive self-concept (Madonna et al., 1990) and are more likely to exhibit adaptive psychological and behavioral adjustment (Roeser et al., 1996). Similarly, classrooms that promote achievement are characterized by high satisfaction, cohesiveness and low friction, without excessive competition (Fraser, 1984; Fraser and O’Brien, 1985). Such an orderly, low-friction environment may reduce opportunities for peer conflict. However, the pathways linking disciplinary climate to school bullying remain under examined and warrant further empirical investigation.

### 1.2 Mediating role of school belonging and school exclusion

School belonging refers to the extent of students’ sense of being accepted, respected, tolerated and countenanced in the school environment (Goodenow, 1993). According to the socio-ecological framework of school belonging, students’ sense of belonging is influenced by their interactions with parents, teachers and peers within the microsystem (Allen et al., 2016), with teacher support identified as a particularly influential factor (Brewster and Bowen, 2004). Students who feel that they receive strong support from their teachers may perceive a sense of warmth, respect, and care within the school environment, which can enhance their sense of belonging to the school (Guo et al., 2024). Numerous studies consistently highlight the important role of teacher support in fostering students’ sense of school belonging. A meta-analysis revealed that among contextual factors, teacher support was the strongest factor of school belonging, even more influential than peer or parental support (Allen et al., 2018). Raufelder and Kulakow (2022) reported that greater teacher support was associated with stronger social belonging and reduced learned helplessness in adolescents. Evidence from China further showed that students who felt more supported by their teachers tended to report a stronger sense of belonging, which was linked to fewer bullying experiences (Guo and Zhao, 2019). Although most research has conceptualized teacher support as a global construct, some studies have begun to distinguish between different types of teacher behavior and have found that supportive practices and punitive disciplinary approaches are differentially associated with students’ sense of school connectedness (Day et al., 2016). The present study also distinguishes between different dimensions of teacher support, specifically focusing on emotional and instructional support, in order to examine their respective influences on students’ sense of school belonging. Given that the outcome variable of the present study is school bullying victimization, the following discussion focuses primarily on how school belonging and school exclusion may shape students’ vulnerability to being bullied, rather than on bullying perpetration.

In addition to teacher support, the perceived disciplinary climate also shapes students’ sense of belonging. Prior studies have indicated that school social environments characterized by clear and fair norms promote students’ civic behaviors and strengthen their emotional connection to school (Lenzi et al., 2014; Resh and Sabbagh, 2017). Similarly, schools with clear and fair disciplinary structures are associated with higher engagement because they cultivate a sense of belonging among students (Guillaume et al., 2015; Konold et al., 2014). Overall, these findings suggest that the emotional and instructional qualities of teacher–student interactions, together with the organizational facet of disciplinary climate, jointly cultivate students’ sense of belonging to school.

Building on this antecedent evidence, a strong sense of school belonging fosters positive emotions and enhances students’ life satisfaction and overall well-being (Xu and Fang, 2021). Empirical evidence indicates that school belonging mediates the impact of academic stress and depression (Liu and Lu, 2012) and buffers against the negative effects of bullying (Li et al., 2020). In fact, school belonging has also been found to influence students’ experiences of bullying victimization (Arslan, 2022). According to the belongingness hypothesis (Baumeister and Leary, 1995), a strong sense of school belonging may serve as a protective factor against external threats, with bullying as one such threat (Guo et al., 2024). Several studies have highlighted the mediating role of school belonging in the relationship between teacher-related factors and bullying victimization, indicating that students with a stronger sense of belonging are less likely to be bullied (Doumas and Midgett, 2019; Jiang et al., 2023; Guo et al., 2024).

While school belonging has received much scholarly attention, far less research has explored the role of school exclusion, which is defined as students’ subjective feeling of being ostracized or overlooked by peers or social groups, difficulties in forming and maintaining interpersonal relationship, and unmet needs for social connection and belonging (Riva and Eck, 2016). Unlike belonging, which reflects social inclusion, exclusion involves perceived rejection and ostracism (Baumeister and DeWall, 2005; Naylor et al., 2006) and has been associated with feelings of loneliness and a general dislike of school (Ramm et al., 2006). From an integrated perspective, exclusion can be understood simultaneously as a structural position within peer hierarchies and a psychological experience of threatened belonging. Peer systems are inherently hierarchical, and bullying reflects asymmetrical power relations within group contexts (Salmivalli, 2010). Students who perceive themselves as excluded are more likely to occupy peripheral positions characterized by limited social support, which may increase their vulnerability to victimization. At the psychological level, social exclusion threatens fundamental needs for belonging and self-worth (Williams, 2007). In line with social identity theory (Tajfel and Turner, 1979), group membership constitutes a central component of self-concept; thus, low belonging or perceived rejection may undermine self-esteem and heighten sensitivity to social threats. Such heightened stress responses (Raufelder and Kulakow, 2022) may further increase susceptibility to bullying. Moreover, bullying should be viewed as a dynamic transactional process rather than a one-sided event (Pellegrini and Long, 2002). Students experiencing exclusion may respond with social withdrawal or defensive behaviors, which can unintentionally reinforce their marginal status and trigger negative peer reactions. In this way, exclusion functions as a more proximal predictor of bullying victimization, whereas belonging represents a broader structural condition shaping peer integration. While empirical studies on the role of school exclusion in bullying victimization are limited, emerging evidence suggests a potential link. Demol et al. (2020) reported that supportive teacher–student relationship was associated with lower levels of peer rejection, which in turn was linked to reduced victimization. Similarly, Liu and Guo (2024) showed that perceived campus exclusion was significantly associated with bullying-related experiences among junior high school students in China, suggesting that exclusion may be relevant to broader bullying processes. In the present study, however, we focus specifically on bullying victimization and interpret school exclusion as a proximal factor that may increase students’ vulnerability to being bullied.

Building on this line of research, the present study integrates school belonging and school exclusion into a unified framework to examine their mediating roles, linking three core domains of teacher–student relationship (teacher emotional support, teacher instructional support and disciplinary climate) to bullying victimization.

Moreover, school belonging and school exclusion are closely related psychological experiences. As Shilling and Brown (2016) suggested, when people experienced social exclusion, they tended to shift their psychological resources toward restoring their fundamental need for belonging. Supporting this view, Li et al. (2017) found that people with a stronger need to belong were more likely to engage in prosocial behaviors after being excluded, whereas Zhang et al. (2020) reported that children with high belonging needs might even adopt group-beneficial deceptive behaviors in response to exclusion. These findings indicate that school belonging and exclusion are interconnected processes, justifying our examination of their serial mediating roles in the pathways from teacher emotional support, teacher instructional support and disciplinary climate to bullying victimization in the present study.

### 1.3 Present study

Although previous research has linked teacher support to students’ bullying victimization, the focus has been predominantly on teacher emotional support (Kim et al., 2021; Thornberg et al., 2024; Guo et al., 2024), while teacher instructional support has received comparatively less attention despite its potential role in enhancing students’ academic confidence and engagement. In addition, the organizational facet of teacher–student interactions, reflected in the clarity and fairness of the disciplinary climate, has rarely been integrated alongside teacher support when explaining pathways to bullying. Existing studies have also tended to emphasize the mediating role of positive socioemotional experiences, such as school belonging (Guo et al., 2024; Guo and Zhao, 2019), whereas the contribution of negative experiences, particularly perceived school exclusion, remains underexamined.

Guided by the Teaching Through Interactions (TTI) framework (emotional, instructional and organizational domains) and social-ecological perspectives on belonging, the present study constructs a serial mediation model in which teacher emotional support (TES), teacher instructional support (TIS), and disciplinary climate are jointly examined in relation to school bullying victimization (SBV) via two interrelated socioemotional mediators, school belonging (SB) and school exclusion (SE).

Accordingly, following hypotheses are proposed:

*H1a*: Teacher emotional support is indirectly negatively associated with students’ bullying victimization through belonging and/or exclusion.

*H1b*: Teacher instructional support is indirectly negatively associated with students’ bullying victimization through belonging and/or exclusion.

*H1c*: A clear and fair disciplinary climate is indirectly negatively associated with students’ bullying victimization through belonging and/or exclusion.

*H2*: School belonging is negatively associated with school exclusion, forming a sequential pathway linking teacher-related factors to bullying victimization.

*H3*: The three domains of teacher–student relationship exhibit differentiated indirect pathways to bullying victimization.

**Figure 1:**
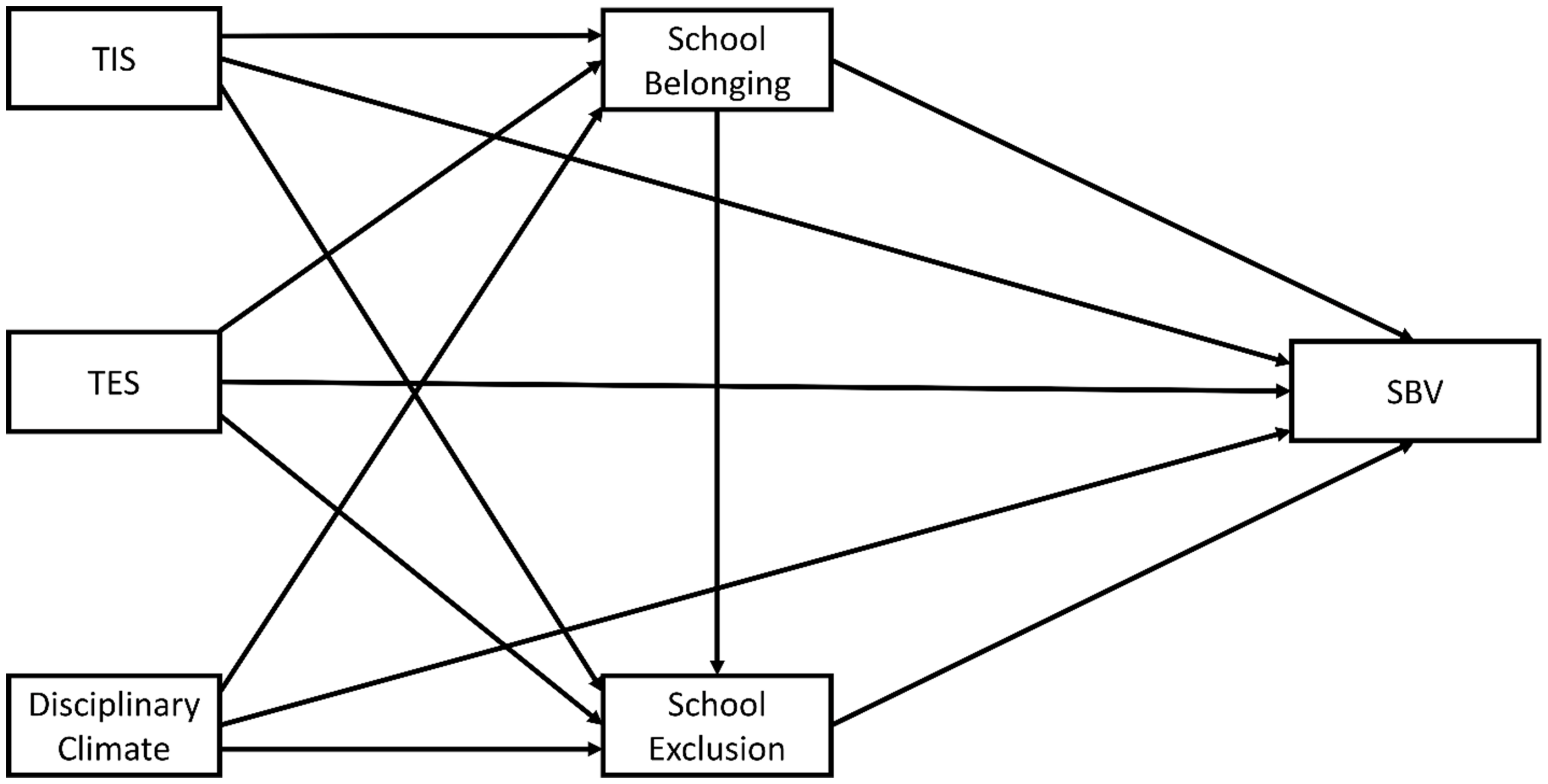
Hypothesized structural model.

The hypothesized model is illustrated in Figure 1.

## 2 Materials and methods

### 2.1 Participants

A convenience cluster sampling method was used to recruit participants from seven middle schools in Wenzhou, China. After excluding invalid or incomplete responses, 798 valid questionnaires were obtained (50.5% male, 49.5% female). This sampling strategy allowed the present study to examine teacher–student relational mechanisms within a relatively homogeneous local educational context, enabling a focused test of the proposed theory-driven mediation.

### 2.2 Measures

The study utilized measurement items from the PISA 2018 student questionnaire administered by the OECD. The PISA 2018 involved extensive pilot testing, item analysis, and expert validation across countries, ensuring strong construct validity and reliability for variables such as bullying victimization, school belonging, and teacher support (OECD, 2019a). The use of these standardized instruments improved the cross-cultural comparability and psychometric quality of the findings in the current Chinese educational context (OECD, 2019b). All scales used a 4-point Likert-type response format, and higher scores reflected stronger endorsement of the construct.

#### 2.2.1 Teacher–student relationship

In this study, teacher–student relationship was framed as a three-dimensional construct comprising emotional support, instructional support, and disciplinary climate, consistent with prior research about secure relationship between teacher and student and theoretical frameworks on teacher–student interactions (Hamre et al., 2013). To assess this construct, we selected 12 items from the PISA 2018 student questionnaire (ST100, ST211 and ST097).

Based on theoretical reasoning and empirical precedent, the items were categorized into three subdimensions: teacher instructional support (4 items; e.g., “The teacher provides extra help when students need it”), teacher emotional support (3 items; e.g., “The teacher shows interest in every student’s learning”) and disciplinary climate (5 items; e.g., “Students do not listen to what the teacher says”). The items of the disciplinary climate subscale were reverse-coded so that higher scores indicate a more positive classroom climate. The responses were rated on a four-point Likert scale (1 = strongly disagree, 4 = strongly agree).

The internal consistency for the subscales was high, with Cronbach’s alpha coefficient of 0.920 for teacher instructional support, 0.911 for teacher emotional support and 0.940 for disciplinary climate.

#### 2.2.2 School belonging and exclusion

We assessed school belonging using six items from the PISA 2018 student questionnaire (ST034). Given the poor fit of a unidimensional model (CFI < 0.90) and consistent with previous research, a two-factor confirmatory factor analysis (CFA) was applied to capture school belonging (3 items; e.g., “I make friends easily at school”) and school exclusion (3 items; e.g., “I feel like an outsider at school”). The responses were recorded on a 4-point Likert scale (1 = strongly disagree to 4 = strongly agree), with higher scores indicating stronger belonging or exclusion. This two-factor structure has been validated in prior PISA-based studies (Raufelder and Kulakow, 2022), reflecting the multidimensional nature of school belonging. In our sample, internal consistency was acceptable (Cronbach’s *α* = 0.786 for school belonging; Cronbach’s *α* = 0.835 for school exclusion).

#### 2.2.3 School bullying victimization

Bullying victimization was measured using six items from the PISA 2018 questionnaire (ST038), which assessed how often students experienced bullying at school (e.g., “Other students left me out on purpose”). The responses were given on a four-point scale (1 = never or almost never; 4 = once a week or more). The scale showed good reliability (Cronbach’s *α* = 0.887).

### 2.3 Procedure

Data collection took place in May 2025. Ethical approval was obtained from the Ethics Committee of Wenzhou University (WZU-2025-033). The study was conducted in accordance with the ethical standards of the institutional and national research committees and with the 1964 Declaration of Helsinki and its later amendments.

Trained research assistants administered paper-and-pencil questionnaires during scheduled class breaks. Participation was voluntary, no incentives were provided, and students could withdraw at any time. For minors, written informed consent was obtained from both the students and their parents or legal guardians. Standardized instructions emphasized independent responding, anonymity, and spatial separation among students to minimize social desirability bias. All completed questionnaires were sealed and collected immediately after completion.

### 2.4 Data analysis

All analyses were conducted in R 4.4.0. Descriptive statistics (means and standard deviations) were calculated based on composite scores (i.e., averaged item scores) to describe sample characteristics. However, all hypothesized relationships were tested using latent variables within a structural equation modeling (SEM) framework to account for measurement error.

Confirmatory factor analysis (CFA) was first conducted using the *lavaan* package to evaluate the measurement model. Model fit was assessed using multiple indices, including CFI, TLI, RMSEA, and SRMR.

Structural equation models were then estimated using maximum likelihood estimation with 5,000 bootstrap samples to obtain robust parameter estimates and bias-corrected confidence intervals for indirect effects.

To assess common method bias, Harman’s single-factor test was performed. The first unrotated factor accounted for 31.0% of the total variance, below the conventional 40% threshold, suggesting that common method variance was unlikely to be a serious concern (Podsakoff et al., 2003).

## 3 Results

### 3.1 Item analysis

The internal and external validity of the measurement items were systematically examined. Regarding internal validity, standardized factor loadings below 0.50 were used as the criterion for item deletion. The results indicated that all standardized factor loadings exceeded the recommended threshold of 0.50, ranging from 0.71 to 0.90 (see Table 1). Therefore, no items were removed at this stage.

**Table 1:** Standardized factor loadings and first-order CFA model fit indices (*N =* 798).

Index	Item	*λ*	χ^2^/df	CFI	TLI	RMSEA	SRMR
TIS	C1	0.84	4.14	0.981	0.975	0.063	0.021
	C2	0.89					
	C3	0.87					
	C4	0.85					
TES	D1	0.86					
	D2	0.89					
	D3	0.90					
DC	E1	0.81					
	E2	0.89					
	E3	0.89					
	E4	0.89					
	E5	0.87					
SB	F2	0.82	2.87	0.992	0.985	0.048	0.022
	F3	0.71					
	F5	0.71					
SE	F1	0.81					
	F4	0.80					
	F6	0.78					
SBV	G1	0.73	15.42	0.948	0.913	0.134	0.035
	G2	0.79					
	G3	0.80					
	G4	0.74					
	G5	0.76					
	G6	0.74					

Subsequently, first-order confirmatory factor analyses (CFA) were conducted for all constructs to evaluate the measurement model. Teacher–student relationships were modeled as three factors: teacher instructional support (TIS), teacher emotional support (TES), and disciplinary climate (DC), while school-related constructs were represented by a two-factor structure of school belonging (SB) and school exclusion (SE), and school bullying victimization (SBV) was treated as a single factor.

The results indicated satisfactory model fit across all constructs (see Table 1). Overall, CFI and TLI values exceeded the recommended threshold of 0.90, and SRMR values were below 0.05, indicating good model fit. Although the RMSEA value for SBV was relatively high, this may be attributed to the low degrees of freedom and the simplicity of the single-factor structure. The overall fit indices supported the adequacy of the measurement models.

All standardized factor loadings were statistically significant (*p <* 0.001) and ranged from 0.81 to 0.90, supporting adequate convergent validity and the reliability of *the* latent constructs.

To examine the internal consistency of the constructs, the Cronbach’s *α* value of each construct was tested and should be over 0.7 (Jackson et al., 2009). Table 2 shows that all Cronbach’s *α* values ranged from 0.79 to 0.94, indicating that the internal reliability of each construct was well established.

**Table 2:** Reliability and validity analysis (*N =* 798).

Items	M	SD	*α*	CR	AVE
1. TIS	3.326	0.736	0.920	0.921	0.744
2. TES	3.306	0.744	0.911	0.912	0.777
3. DC	3.438	0.699	0.940	0.939	0.756
4. SB	2.895	0.751	0.786	0.790	0.557
5. SE	1.828	0.785	0.835	0.835	0.628
6. BV	1.367	0.590	0.887	0.891	0.578

Moreover, the composite reliability (CR) of each construct was examined to assess external consistency. As presented in Table 2, the CR values ranged from 0.79 to 0.94, further demonstrating satisfactory external reliability for all constructs.

To examine the convergent validity, the average variance extracted (AVE) for each construct should exceed the recommended threshold of 0.50 (Jackson et al., 2009). As shown in Table 2, the AVE values ranged from 0.557 to 0.777, indicating that all constructs demonstrated adequate convergent validity. These results suggest that the indicators of each construct shared a high proportion of variance in common.

Furthermore, following the recommendation of Awang (2015), discriminant validity was assessed by comparing the square root of the AVE for each construct with the correlation coefficients among constructs. The square root of the AVE for each construct was greater than the corresponding inter-construct correlations (see Table 3), supporting satisfactory discriminant validity. These findings indicate that each construct was empirically distinct from the others.

**Table 3:** Construct discriminant validity analysis (*N =* 798).

Construct	1	2	3	4	5	6
1. TIS	**0.863**					
2. TES	0.817***	**0.882**				
3. DC	0.351***	0.269***	**0.869**			
4. SB	0.450***	0.468***	0.171***	**0.745**		
5. SE	−0.400***	−0.375***	−0.505***	−0.539***	**0.792**	
6. BV	−0.341***	−0.317***	−0.350***	−0.410***	0.542***	**0.762**

***p < 0.001. Bold values on the diagonal are the square roots of AVE. To establish the discriminative validity, the value should be greater than the inter-construct correlations.

### 3.2 Descriptive profile of bullying victimization

Although school bullying victimization was retained as a single latent construct in the main SEM, additional item-level descriptive analyses were conducted to characterize the frequency and type of bullying victimization in the present sample. Because the PISA bullying items assess occurrence frequency rather than formally graded severity, students were not classified into mild or severe victimization groups. Instead, repeated/frequent victimization was defined as reporting at least one relevant item as occurring a few times a month or more.

As shown in Tables 4, 165 students (20.7%) reported repeated/frequent general bullying victimization. Type-specific analyses indicated that verbal bullying victimization was the most frequently reported form (*n =* 109, 13.7%), followed closely by relational bullying victimization (*n =* 108, 13.5%). Physical bullying victimization was less frequently reported (*n =* 79, 9.9%). These findings suggest that bullying victimization in the present sample involved multiple behavioral forms, with verbal and relational victimization being slightly more common than physical victimization. Thus, the descriptive analysis provides a frequency-based characterization of repeated/frequent victimization and its behavioral forms, while the main SEM retained bullying victimization as a continuous latent construct.

**Table 4:** Prevalence of general and type-specific bullying victimization (*N =* 798).

Type of bullying victimization	Items	*n*	%
General bullying victimization	g1–g6	165	20.7
Relational bullying victimization	g1, g6	108	13.5
Verbal bullying victimization	g2, g3	109	13.7
Physical bullying victimization	g4, g5	79	9.9

Bullying victimization was coded as present when a student reported at least one corresponding act occurring a few times a month or more. General bullying victimization was coded as present when this criterion was met for at least one of the six victimization items. Type-specific categories were not mutually exclusive.

### 3.3 Model fit analysis

Structural equation modeling (SEM) was conducted to examine the structural relationships among teacher instructional support (TIS), teacher emotional support (TES), disciplinary climate (DC), school belonging (SB), school exclusion (SE), and school bullying victimization (SBV). The hypothesized Model incorporated parallel mediation (via SB and SE), a serial pathway, and direct effects from all TTI factors to victimization. To control for potential collinearity between the three independent variables, a covariance path was specified between them.

The overall fit indices indicated that the proposed model demonstrated a good fit to the data. The chi-square statistic was significant, *χ*^2^ (237) = 696.36, *p <* 0.001, which is expected given the large sample size. The relative chi-square (*χ*^2^/df = 2.94) fell below the recommended threshold of 3.00, indicating acceptable fit. Incremental fit indices exceeded recommended standards (CFI = 0.966; TLI = 0.960; IFI = 0.966; RFI = 0.940), all above the conventional criterion of 0.90. The absolute fit indices also suggested satisfactory model fit (GFI = 0.991; AGFI = 0.988, RMSEA = 0.049, SRMR = 0.032), further supporting good model adequacy. Overall, the fit statistics indicate that the hypothesized structural model provides an adequate and robust representation of the observed data.

### 3.4 Mediating effects of school belonging and school exclusion

**Figure 2:**
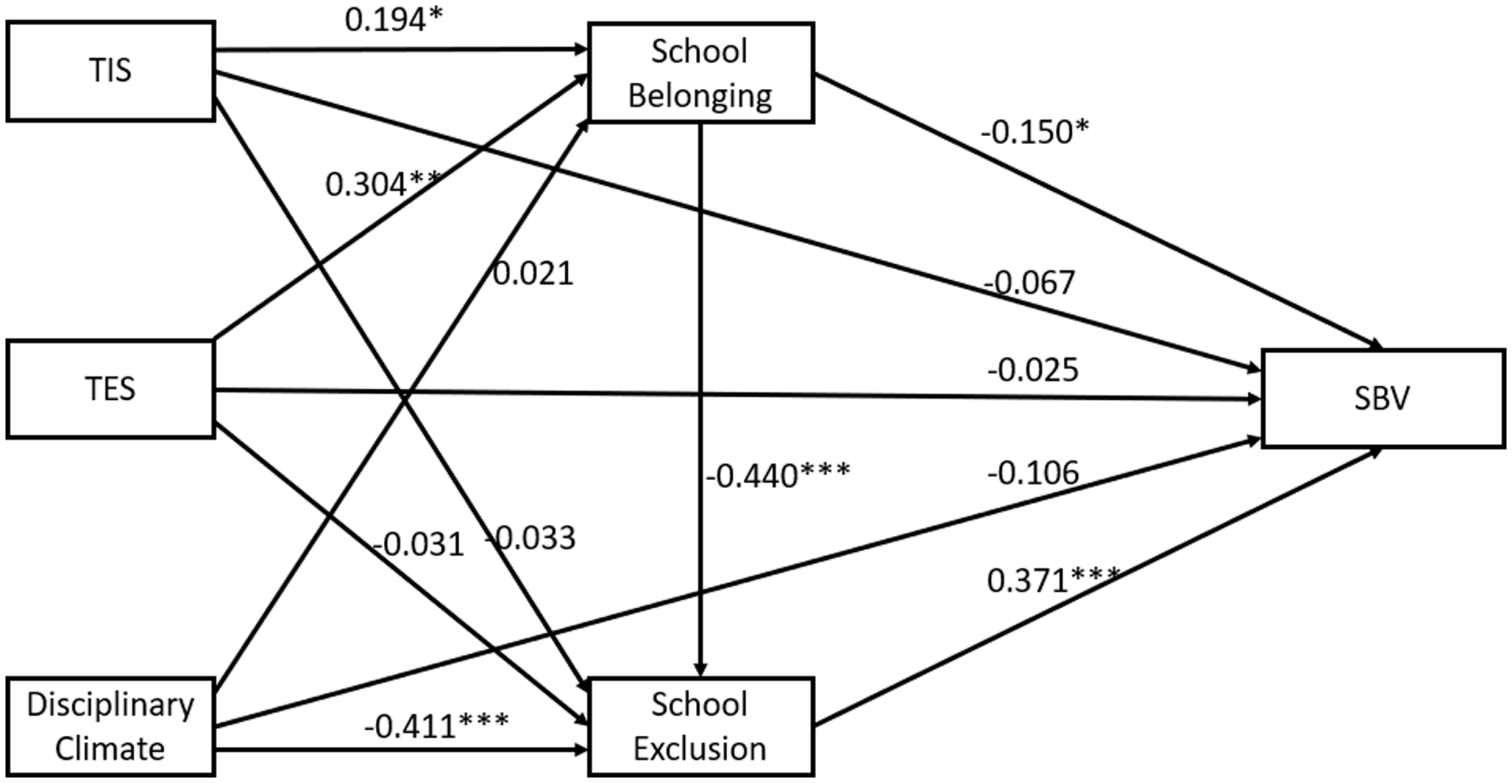
Mediating model of school belonging and school exclusion. *p < 0.05, **p < 0.01, ***p < 0.001.

The parameter estimates shown in Figure 2 indicate that TIS (*β* = 0.194, *p* = 0.032) and emotional support (*β* = 0.304, *p* = 0.001) were positively associated with school belonging. Disciplinary climate was not significantly associated with belonging (*β* = 0.021, *p* = 0.645). School exclusion was negatively associated with disciplinary climate (*β* = −0.411, *p <* 0.001) and school belonging (*β* = −0.440, *p <* 0.001). The paths from instructional support and emotional support to exclusion were not statistically significant. Bullying victimization was negatively associated with school belonging (*β* = −0.150, *p* = 0.025) and positively associated with school exclusion (*β* = 0.371, *p <* 0.001). After inclusion of the mediators, the direct paths from instructional support (*β* = −0.067, *p* = 0.359), emotional support (*β* = −0.025, *p* = 0.726), and disciplinary climate (*β* = −0.106, *p* = 0.121) to victimization were not statistically significant.

The model accounted for 23.3% of the variance in school belonging (R^2^ = 0.23), 47.0% in school exclusion (R^2^ = 0.47), and 33.1% in bullying victimization (R^2^ = 0.33).

To evaluate the contribution of the mediating variables, a reduced model excluding the paths from belonging and exclusion to victimization was estimated. In the reduced model, the explained variance in victimization decreased from R^2^ = 0.33 to R^2^ = 0.19. The corresponding incremental effect size was f^2^ = 0.22, indicating a medium contribution according to Cohen’s (1988) criteria.

When the path from belonging to exclusion was removed, the explained variance in school exclusion decreased from R^2^ = 0.47 to R^2^ = 0.33, yielding a medium incremental effect (f^2^ = 0.27). The corresponding incremental contribution to bullying victimization was small (f^2^ = 0.02).

Bootstrap analyses based on 5,000 bias-corrected and accelerated resamples (see Table 5) demonstrated that teacher instructional support (TIS) exerted a significant indirect association with bullying victimization through school belonging (*β* = −0.036, 95% CI [−0.097, −0.003]). A significant serial pathway was likewise identified, indicating that TIS was indirectly linked to victimization via school belonging and subsequent school exclusion (*β* = −0.039, 95% CI [−0.094, −0.006]). When the full mediating structure was considered, the overall indirect effect of TIS remained significant (*β* = −0.089, 95% CI [−0.187, −0.002]). A comparable but more pronounced pattern emerged for teacher emotional support (TES). TES showed a significant indirect association with victimization through school belonging (*β* = −0.056, 95% CI [−0.137, −0.009]), as well as through the sequential pathway from belonging to exclusion (*β* = −0.061, 95% CI [−0.134, −0.025]). Consistent with these specific pathways, the total indirect effect of TES was also significant (*β* = −0.131, 95% CI [−0.249, −0.048]). In contrast, disciplinary climate (DC) operated through a more focused mechanism. A significant indirect effect was observed via school exclusion (*β* = −0.186, 95% CI [−0.303, −0.095]), and the corresponding total indirect effect was likewise significant (*β* = −0.194, 95% CI [−0.304, −0.105]). These findings suggest that instructional and emotional support were associated with victimization primarily through school belonging and its downstream relation to exclusion, whereas disciplinary climate was linked to victimization predominantly through school exclusion.

**Table 5:** Bootstrap indirect effects of teacher-related variables on bullying victimization (5,000 resamples).

Predictor	Indirect path	*β*	95% CI	*p*
TIS	Parallel (SB)	−0.036	[−0.097, −0.003]	0.121
	Parallel (SE)	−0.015	[−0.081, 0.047]	0.646
	Serial (SB–SE)	−0.039	[−0.094, −0.006]	0.075
	Total Indirect Effect	−0.089	[−0.187, −0.002]	0.055
TES	Parallel (SB)	−0.056	[−0.137, −0.009]	0.076
	Parallel (SE)	−0.014	[−0.084, 0.045]	0.653
	Serial (SB–SE)	−0.061	[−0.134, −0.025]	0.024
	Total Indirect Effect	−0.131	[−0.249, −0.048]	0.010
DC	Parallel (SB)	−0.004	[−0.070, 0.010]	0.682
	Parallel (SE)	−0.186	[−0.303, −0.095]	<0.001
	Serial (SB–SE)	−0.004	[−0.070, 0.010]	0.682
	Total Indirect Effect	−0.194	[−0.304, −0.105]	< 0.001

*β*, unstandardized indirect effect. Confidence intervals are bias-corrected and accelerated (BCa) based on 5,000 bootstrap samples. Effects are considered significant when the 95% confidence interval does not include zero.

## 4 Discussion

The present study employed structural equation modeling (SEM) to examine how teacher–student relationship was associated with students’ bullying victimization, with a particular focus on indirect pathways via school belonging and exclusion.

### 4.1 Mediating role of school belonging and school exclusion

Consistent with Hypotheses 1a–1c, the results demonstrated that school belonging and school exclusion functioned as key mediators linking the three domains of teacher–student relationship to school bullying victimization. Drawing on socio-ecological theory (Bronfenbrenner, 1979), the present findings suggest that teacher–student relationship functions as a proximal relational context within the classroom microsystem shaping students’ vulnerability to bullying victimization through layered relational mechanisms. Rather than exerting uniform protective effects, instructional support, emotional support, and disciplinary climate demonstrated differentiated pathways involving belonging and exclusion.

These mechanism-oriented findings are consistent with a broader body of school-climate research showing that teacher-related and classroom-level conditions are closely linked to bullying victimization. Using cross-national PISA 2018 data, the OECD (2019c) reported that teacher fairness and classroom order were consistently associated with lower bullying exposure across countries, yet these associations were largely indirect and embedded within broader perceptions of school climate. Similarly, meta-analytic research has shown that dimensions of school climate, particularly teacher support and classroom organization, are robustly associated with reductions in bullying and peer victimization (Aldridge and McChesney, 2018). Longitudinal evidence further indicates that teacher support predicts lower subsequent victimization through its influence on peer relational processes (Demol et al., 2020). These studies suggest that bullying victimization should be understood not only as an individual experience, but also as an outcome embedded in relational and organizational features of the school microsystem.

Recent large-scale evidence from China has shown that bullying victimization is associated with a broad range of psychological difficulties and has highlighted the relevance of distinguishing different forms of victimization (Zhao et al., 2024). In the present sample, item-level descriptive results showed that repeated/frequent victimization occurred across relational, verbal, and physical forms, with verbal and relational victimization being slightly more common than physical victimization. These descriptive findings contextualize the outcome variable, while the main SEM treats bullying victimization as a general latent construct to examine how teacher-related factors, school belonging, and school exclusion are associated with students’ general vulnerability to victimization.

The pattern of indirect effects further clarifies how the three teacher-related domains operated through distinct socio-relational pathways. Teacher emotional support appeared to function mainly through an affiliative pathway. When teachers were perceived as caring, responsive, and emotionally available, students were more likely to feel accepted and valued within the school community; this stronger sense of belonging, in turn, was associated with lower perceived exclusion and reduced vulnerability to victimization. This interpretation is consistent with prior evidence that emotionally supportive teacher–student relationships foster peer connectedness and reduce rejection-related risks (Van Gils et al., 2023; Demol et al., 2020). By contrast, disciplinary climate seemed to operate more directly through an exclusion-regulation pathway. A fair and orderly classroom climate may reduce opportunities for peer marginalization by making classroom norms clearer and social boundaries less arbitrary, which helps explain why school exclusion was a particularly important mediator in this pathway. Instructional support showed a weaker but still meaningful indirect pathway, suggesting that well-structured and supportive instruction may promote students’ sense of competence and participation in classroom life, thereby strengthening school belonging and indirectly reducing exclusion-related vulnerability. These differentiated pathways suggest that teacher–student relationship affects bullying victimization not through a single generalized protective process, but through domain-specific mechanisms of emotional connection, classroom norm regulation, and instructional inclusion.

This pattern also helps explain why the direct paths from teacher-related variables to victimization were no longer statistically significant once belonging and exclusion were introduced into the model. Rather than indicating the absence of teacher influence, these results suggest that the associations between teacher-related conditions and bullying victimization were largely accounted for by students’ socio-emotional integration within the classroom microsystem.

### 4.2 Differentiated mechanisms within the TTI framework

Consistent with attachment perspectives (Verschueren and Koomen, 2012), emotional support was strongly associated with school belonging. Teachers who are emotionally responsive may function as secondary attachment figures, fostering students’ sense of security and acceptance. Recent longitudinal evidence further supports this relational pathway. Van Gils et al. (2023) found that perceived teacher emotional support predicted decreases in peer victimization over time, primarily through enhanced peer connectedness. These findings align with contemporary belonging research emphasizing belonging as a structural integrative force within peer networks rather than merely an affective state (Allen et al., 2018). The significant serial pathway suggests that emotional support reduces victimization risk through sequential socio-emotional processes involving both belonging and exclusion.

In contrast, disciplinary climate operated predominantly through school exclusion rather than belonging. This distinction resonates with the TTI framework (Hamre et al., 2013), which conceptualizes classroom organization as structurally distinct from emotional support. Multilevel research has shown that classroom-level norms and rule enforcement are significantly associated with reductions in bullying and victimization (Saarento et al., 2015), suggesting that structured classroom management may constrain the social conditions under which exclusionary interactions emerge. In line with this structural interpretation, disciplinary climate in the present study was strongly linked to exclusion, which in turn predicted victimization. This pattern suggests that disciplinary climate may regulate the structural opportunities under which exclusionary interactions occur, rather than directly shaping students’ sense of relational security.

Instructional support demonstrated a weaker but still meaningful indirect association with victimization through belonging and the subsequent belonging–exclusion sequence. Although instructional support was not directly associated with victimization after accounting for mediators, the significance of the indirect pathway suggests that its protective influence depends on relational embedding within the peer ecology. This pattern is consistent with recent findings suggesting that instructional support may operate through internal psychological mechanisms on victimization rather than direct social pathways (Zeng et al., 2023). The comparatively smaller indirect effects observed here suggest that cognitive scaffolding may contribute to victimization prevention only insofar as it strengthens students’ psychological resources like competence and engagement that are subsequently translated into relational integration. Rather than relying on a singular pathway, its influence appears to emerge from the complementary accumulation of belonging-based and sequential mechanisms.

Consistent with Hypothesis 3, the pattern of findings suggests that teacher–student relationship dimensions are associated with victimization through differentiated relational pathways within the TTI framework. Emotional support functions as a relational catalyst, contributing to sequential processes involving belonging and exclusion that ultimately relate to victimization. This interpretation is consistent with prior studies showing that emotionally supportive teacher–student relationships foster students’ peer connectedness and reduce rejection-related risks (Van Gils et al., 2023; Demol et al., 2020). Instructional support operates in a more distributed manner, exerting influence through the cumulative integration of multiple indirect routes rather than a singular dominant pathway. This aligns with the TTI framework, in which instructional support is understood as shaping students’ engagement, participation, and classroom integration rather than only providing academic assistance (Hamre et al., 2013). Disciplinary climate, in contrast, represents a structural regulator, directly shaping the exclusionary conditions that give rise to victimization risk. This finding is also supported by school-climate research indicating that fair classroom norms, teacher fairness, and classroom order are associated with lower levels of peer victimization (OECD, 2019c; Aldridge and McChesney, 2018). Together, these differentiated mechanisms suggest that teacher–student relationship factors relate to victimization through partially distinct relational and structural channels.

### 4.3 Belonging–exclusion as a coupled regulatory system

A key theoretical contribution of this study lies in modeling belonging and exclusion as dynamically coupled constructs rather than conceptual opposites. Consistent with Hypothesis 2, the significant path from belonging to exclusion supports a sequential interpretation in which positive integration is associated with lower levels of marginalization. Empirical evidence further suggests that school connectedness is systematically linked to social rejection processes. Shochet et al. (2006) demonstrated that lower school belonging was associated with higher levels of peer rejection, underscoring the structural interdependence between inclusion and marginalization dynamics. Recent longitudinal evidence further highlights the broader regulatory role of school belonging in adolescent adjustment, demonstrating that belonging buffers the psychological consequences of victimization over time (Arslan and Allen, 2021).

Removing the belonging-to-exclusion path substantially reduced explained variance in exclusion (f^2^ = 0.27), underscoring belonging’s foundational role within peer ecology. However, its incremental contribution to victimization was comparatively small (f^2^ = 0.02), indicating that exclusion serves as the more proximal predictor of victimization.

This layered configuration aligns with well-established peer victimization research distinguishing relational marginalization from overt victimization processes (Hawker and Boulton, 2000; Reijntjes et al., 2010). Importantly, school exclusion in the present model should not be interpreted as a subtype of bullying victimization. Rather, it refers to a broader socio-relational condition of marginalization and weak peer integration that may increase vulnerability across different behavioral forms of victimization.

Beyond statistical significance, effect size analyses provide insight into explanatory strength. The mediating system accounted for a medium incremental contribution to victimization (f^2^ = 0.22), as the explained variance decreased from R^2^ = 0.33 to R^2^ = 0.19 when belonging and exclusion were removed. This magnitude suggests that the explanatory power of the relational mechanism is not trivial but represents a substantively meaningful component of victimization risk. Contemporary prevention science increasingly emphasizes mechanism-driven intervention rather than direct symptom suppression (Gaffney et al., 2019). The present findings quantitatively reinforce this shift, demonstrating that teacher–student relationship shapes victimization risk primarily through regulation of socio-emotional integration processes.

### 4.4 Theoretical and practical implications

The present findings have both theoretical and practical implications for understanding and preventing bullying victimization. Theoretically, these results integrate socio-ecological, attachment-based, and classroom interaction frameworks into a unified multistep mechanism model. The differentiated pathways observed suggest that relational and structural classroom processes operate through partially distinct channels.

Practically, the present findings suggest that bullying prevention should not be limited to general calls for stronger teacher support, greater school belonging, or better classroom management. Rather, schools should adopt mechanism-informed strategies that correspond to the differentiated pathways identified in the model. Given the cross-sectional design of this study, the following recommendations should be interpreted as practical implications rather than causal prescriptions.

First, given the strong association between emotional support and school belonging, teachers may strengthen students’ sense of being noticed, accepted, and supported through stable, low-intensity daily interactions. For example, homeroom teachers or subject teachers could conduct brief but regular check-ins with students who appear socially isolated, are frequently absent, show reluctance to participate in classroom activities, or repeatedly become targets in peer interactions. These check-ins do not need to take the form of formal counselling; rather, they may involve teacher-initiated conversations that communicate recognition, concern, and the availability of support. Teachers should also avoid interactional patterns in which sustained positive attention is given mainly to academically high-performing or socially active students, and should deliberately recognize students who are less visible in classroom interactions. In this way, emotional support can operate more specifically through students’ relational security and sense of school belonging.

Second, schools should treat exclusionary peer processes as early targets of bullying prevention. Because school exclusion emerged as the more proximal predictor of bullying victimization, interventions should not focus only on overt bullying incidents after they have occurred, but should also address the relational conditions through which students are gradually pushed to the margins of the peer group. In practice, teachers may use simple classroom observation records to monitor repeated social isolation, refusal to include a student in collective activities, mocking, rumor spreading, deliberate ignoring, online exclusion, or bystander reinforcement. These behaviors should not be dismissed as ordinary peer conflict, because they may signal the relational conditions under which bullying victimization becomes more likely. Accordingly, disciplinary climate should move beyond general behavioral control toward the explicit naming and consistent interruption of exclusionary interactions. Teachers and students may jointly formulate classroom norms that address relational aggression, social exclusion, humiliating jokes, rumor spreading, and bystander encouragement, and these norms should be enforced consistently and fairly across students. At the same time, disciplinary responses should avoid public humiliation, as humiliating punishment may unintentionally reproduce the exclusionary dynamics that prevention efforts seek to reduce. In this sense, disciplinary climate functions not merely to maintain classroom order, but to reduce the social opportunities through which exclusionary interactions persist and escalate.

Third, instructional support should be designed as a mechanism for inclusive classroom participation, not merely as a means of improving academic performance. Although the indirect role of instructional support was comparatively weaker in the present model, its significant pathway suggests that instructional organization may still shape peer relations by increasing students’ competence and visibility in classroom activities. Teachers can provide differentiated scaffolding, clear task structures, tiered task goals, and low-threat feedback to help low-achieving students, quiet students, or students at the margins of peer networks experience visible success. In cooperative tasks, teachers may assign and rotate roles such as recorder, presenter, summarizer, or materials coordinator, thereby preventing dominant students from monopolizing participation and reducing the likelihood that academically weaker students are perceived as burdensome group members. Classroom evaluation should also minimize public comparison and labeling feedback, so that academic differences are not converted into peer status hierarchies. Thus, instructional support should be understood not only as cognitive scaffolding, but also as a classroom mechanism shaping whether students are perceived by peers as competent, contributing, and legitimate members of the classroom community, thereby complementing emotional support and disciplinary climate in a broader mechanism-informed approach to bullying prevention.

### 4.5 Limitations and future directions

Despite its contributions, several limitations should be acknowledged. The sample was drawn from a limited number of schools in eastern China, which may constrain the generalizability of the results to other regions and educational contexts. Replication with larger and more diverse samples would strengthen the external validity. In addition, all constructs were measured through student self-reports, raising the possibility of shared method variance; future work could incorporate multiple informants or observational measures. Finally, the cross-sectional design precludes conclusions about causality. Alternative directional configurations cannot be ruled out in cross-sectional designs. Longitudinal or experimental approaches are essential for tracing developmental trajectories and clarifying the mechanisms linking teacher–student relationship, belonging, exclusion, and bullying victimization. Although bullying victimization in the present sample varied in both frequency and behavioral form, it was modeled as a general latent construct in the main analysis. This approach was consistent with the study’s primary aim of examining broad relational mechanisms linking teacher-related factors, school belonging, school exclusion, and victimization. Future research may further examine whether these relational mechanisms differ across levels of victimization frequency or across relational, verbal, and physical forms of bullying victimization. Future research could also examine whether the relational mechanisms identified in the present study vary by gender, particularly given that boys and girls may differ in their exposure to different behavioral forms of bullying victimization. In addition, the present study focused specifically on bullying victimization and did not examine whether school belonging and school exclusion may play similar mediating roles in relation to bullying perpetration. Future research could address this issue by distinguishing more clearly between victimization and perpetration outcomes.

## 5 Conclusion

This study conceptualized teacher–student relationship as a relational mechanism embedded within the classroom microsystem and examined how multiple dimensions of teacher support and disciplinary climate relate to bullying victimization through school belonging and school exclusion. The findings indicate that teacher-related factors are not directly linked to victimization; rather, their influence operates primarily through socio-emotional integration processes within the peer ecology.

Specifically, emotional and instructional support were associated with enhanced school belonging, whereas disciplinary climate was primarily associated with reduced school exclusion. Belonging and exclusion functioned as dynamically coupled constructs, with stronger belonging relating to lower exclusion and, in turn, to reduced bullying victimization. This layered configuration suggests that relational security and structural classroom conditions jointly regulate vulnerability to peer victimization.

By integrating socio-ecological, attachment-based, and Teaching Through Interactions perspectives, the present study advances a multistep explanatory model in which teacher practices shape victimization risk through partially distinct relational and structural channels. These findings underscore the importance of targeting socio-emotional integration processes in bullying prevention by strengthening school belonging as a foundational relational resource that may subsequently reduce school exclusion, which in turn represents a more proximal predictor of victimization. Rather than relying solely on broad anti-bullying messages or behavioral control strategies, effective prevention may benefit from mechanism-informed practices that combine emotionally responsive teacher interactions, early identification and interruption of exclusionary peer processes, and inclusive instructional organization that increases students’ competence and visibility within the classroom community.

## Data Availability

The raw data supporting the conclusions of this article will be made available by the authors, without undue reservation.
